# Chinese small and medium-sized city image communication on Douyin: Algorithmic heuristics and cultural resilience in short-video ecologies

**DOI:** 10.1371/journal.pone.0354468

**Published:** 2026-07-23

**Authors:** Feixiang Li, Xueqin Peng

**Affiliations:** School of Literature, Journalism & Communication, Guangdong Ocean University, Zhanjiang, Guangdong Province, China; Dong-A University College of Business Administration, KOREA, REPUBLIC OF

## Abstract

This study examines how Chinese small and medium-sized cities gain visibility and sustain place-based meaning within Douyin’s platformized short-video ecology. Focusing on Shaoguan, Guangdong Province, it analyzes 30 high-interaction Douyin videos published between May 2023 and May 2025 and applies crisp-set Qualitative Comparative Analysis (csQCA) to identify configurations associated with high dissemination performance. The findings suggest that city-related hashtags function as a necessary platform-indexing condition in the sampled archive, while emotional resonance and narrative structure strengthen specific high-impact configurations. Two communicative logics are particularly salient: non-local creators can generate rapid visibility through immersive, fragmented, and affective presentations, whereas locally embedded storytelling can reinforce cultural resilience and place identity. The study contributes to digital urban communication by integrating the Heuristic-Systematic Model with debates on platformization, algorithmic visibility, and digital place branding.

## 1. Introduction

China’s ongoing urbanization has heightened the strategic role of small and medium-sized cities (SMCs) in regional development, cultural continuity, and public communication. At the same time, short-video platforms such as Douyin have transformed city-image communication by embedding urban representation within recommendation systems, creator economies, hashtag publics, and platform-specific attention metrics. Under network media logic, visibility is no longer produced only by institutional messaging; it is co-produced by users, interfaces, algorithms, and circulating cultural cues [1–3]. This makes the study of SMCs on Douyin timely because peripheral and non-metropolitan cities must compete for attention in an environment where discoverability and recognizability are increasingly platform-governed.

Existing research has provided valuable accounts of city branding, multimodal urban representation, and short-video engagement, including recent work on city-image promotion short videos and Chinese urban imaginaries [4–7]. However, two gaps remain. First, many studies treat the city as a destination brand or a communication object, but pay less attention to how platform affordances structure the conditions under which small and medium-sized cities become visible. Second, single-factor explanations cannot fully explain why different combinations of hashtags, creator identity, emotion, narrative, and visual design may lead to similar dissemination outcomes.

This study addresses these gaps by examining Shaoguan, a prefecture-level city in northern Guangdong Province, as a theory-informed case of SMC communication. Shaoguan is analytically useful because it possesses recognizable cultural and ecological resources, including Danxia Mountain, Hakka culture, Nanhua Temple, and local foodways, but does not enjoy the automatic symbolic legibility of China’s major metropolitan centers. The case therefore enables us to ask how local cultural resources are translated into platform-recognizable signals and how such signals interact with deeper narratives of cultural resilience.

The article makes three contributions. Theoretically, it connects the Heuristic-Systematic Model (HSM) with platformization and digital place-branding scholarship to conceptualize a cue-narrative mechanism of Douyin-based city communication. Methodologically, it uses csQCA to model conjunctural causality and equifinality among 30 high-interaction videos. Empirically, it identifies how algorithmic heuristics and cultural narratives combine to produce high dissemination performance, thereby clarifying how SMCs can pursue short-term visibility without reducing place identity to purely traffic-oriented signs.

## 2. Theoretical background

### 2.1 City media image and place branding

Communication has long shaped how urban identities are imagined. Foundational urban semiotics highlights the role of visual and symbolic elements--landmarks, architecture, and spatial legibility--in structuring public imaginaries of the city [8]. Building on this tradition, place-branding scholarship stresses culture, tourism, civic identity, and stakeholder meaning-making as core dimensions of city brands [4,5,9,10]. Tourism and audiovisual communication research similarly shows that place images are constructed through organizational narratives, promotional mediation, and affective destination imaginaries [11–12]. With the diffusion of short-video platforms in China, city branding is increasingly driven by user-generated content and continuous micro-storytelling rather than top-down campaigns: affect-laden cues and everyday scenes travel quickly, shaping audience evaluations and shareability in digital streams [13–14].

### 2.2 Evolution of city image communication

Classical models framed communication as a linear process--‘who says what in which channel to whom with what effect’ [15]--and municipal promotions long relied on broadcast-style videos and print. In today’s China, platformized short-video communication reconfigures production and circulation: algorithmic feeds couple with participatory authorship, shifting from networked publics to interest-clustered publics and from institutional media logic to network media logic [1,3,13,16,17]. Platformization further means that cultural content, creator practices, and datafied governance are reorganized around platform infrastructures and metrics [2]. Chinese evidence suggests that multimodal videos articulate city history/modernity pairings and help municipal actors and creators co-construct attention, with specific content features linked to dissemination outcomes [6–7]. These developments motivate our configurational approach.

### 2.3 Cognitive psychology and the Heuristic–Systematic Model

The Heuristic-Systematic Model (HSM) explains how audiences process persuasive information via low-effort heuristics (e.g., source/format cues) and high-effort systematic processing (content-driven meaning-making) [18–19]. In digital environments, technological affordances trigger recognizable heuristics, while credibility judgments frequently rely on cognitive shortcuts [20–21]. Research on algorithmic visibility further shows that creators and influencers negotiate platform cues, attention economies, and visibility labor rather than merely publishing content into a neutral media space [22–23]. Applied to Douyin, we conceptualize hashtags and cover cues as algorithmic heuristics orienting attention, and emotional resonance and narrative structure as systematic cues sustaining identification. This cue-narrative alignment underpins our case design and variable selection.

### 2.4 Research propositions

Based on the preceding literature, three theoretical propositions guide the analysis.

Proposition 1: In platformized short-video environments, city-related hashtags and visual packaging function as algorithmic heuristics that increase the likelihood of initial visibility for small and medium-sized cities.
Proposition 2: High dissemination is more likely when heuristic cues are combined with systematic cultural resources, especially emotional resonance and narrative structure, because such combinations link rapid recognizability with deeper place meaning.
Proposition 3: Creator positionality shapes how local cultural resources are translated into platform visibility: non-local creators can generate novelty and comparative attention, whereas local creators can strengthen embedded knowledge and cultural resilience.

## 3. Methods

To improve transparency, this section is organized around two linked components: the research procedure and sample, and the research tools used to analyze the archive. The first component explains why Shaoguan and why a bounded set of high-interaction Douyin videos is appropriate for the research question. The second component explains how HSM and csQCA are combined to identify configurational pathways rather than isolated net effects.

### 3.1 Research procedure and sample

The study follows a bounded case-archive design. Shaoguan was selected because it is a Chinese small and medium-sized city with recognizable cultural and ecological resources but comparatively limited national media visibility. The sample consists of 30 high-interaction Douyin videos about Shaoguan published between May 2023 and May 2025. The archive was constructed through purposive, cross-account keyword retrieval using ‘Shaoguan,’ ‘Shaoguan tourism,’ ‘Danxia Mountain,’ and ‘Hakka culture.’ This sampling strategy is not intended to represent all Shaoguan-related videos on Douyin statistically; rather, it captures videos that had already entered the platform’s attention circuit and is therefore appropriate for examining how SMC visibility is configured once dissemination has occurred.

### 3.2 Research tools

Two research tools structure the analysis. First, HSM provides the conceptual distinction between heuristic cues and systematic meaning-making. In China’s short-video ecology, hashtags, covers, creator badges, and duration operate as algorithmic heuristics that orient attention and retrieval, whereas emotional resonance, narrative coherence, and camera language support more systematic interpretation. Second, csQCA provides a set-theoretic tool for identifying necessity, sufficiency, conjunctural causality, and equifinality in a medium-N archive. We implemented the analysis with fsQCA (Windows v4.1) and report necessity, sufficiency, calibration, PRI-consistency, and robustness checks following established QCA guidance [24–25].

### 3.3 Sample documentation and representativeness

To reduce personalization bias, searches were conducted across new accounts, institutional accounts, and UGC accounts. The final archive represents a theoretically relevant subset of Shaoguan videos that achieved relatively high interaction, not a probability sample of all Douyin content. Its representativeness is therefore analytical rather than statistical: the cases reflect the visible segment of Shaoguan’s platform communication and allow comparison across creator identity, hashtag use, narrative form, affective cues, and visual design. This scope also defines the limits of generalization. The findings can inform comparable SMC communication on short-video platforms, but they should not be read as population-level estimates of all city-image videos. The selected cases and case identifiers are listed in Table 1.

**Table 1:** Case selection table.

No.	Case Description	No.	Case Description
C1	Wonton’s Dad/Dancing to Remember My Hometown	C16	Xu Ge Yuan/No Way, Danxia Mountain in Shaoguan Doesn’t Require Tickets?
C2	Los Angeles Ying Zheng/ Journey to Danxia	C17	Guangdong Culture & Tourism, Shaoguan Culture & Tourism/Zhang Songwen Came to Shaoguan, Why Haven’t You?
C3	Liangde Jewelry Gold & Silver Workshop/The Cautious Boss	C18	Niangao Gao/Two Minutes to Record My 5-Hour Climb to the Top of Danxia Mountain
C4	Zhang Yin/Traveling Shaoguan Through Historical Records	C19	Yun Yun Baby/New Year’s Wish to Pray and Check In
C5	History Researcher/Shaoguan Once Ranked as Guangdong’s Second Largest Industrial City	C20	Lin Yizong/Please Don’t Go to Crowded Big Cities During Holidays
C6	Old Huang Arrives in Shaoguan/Returning to My Hometown Today	C21	Heili/Why Are Cantonese People So Obsessed with Farmhouse Dining?
C7	It’s Alan, Not A Lan/Shaoguan Trip Vlog: Popular Restaurant Edition	C22	Ruyuan Media/Head to Ruyuan to Watch the HD Version
C8	Flying Biu/Start a Summer Adventure in Shaoguan Right Away	C23	Xiaohei Zhuming/As Red as Cinnabar, As Bright as Rosy Clouds
C9	Shaoguan Culture & Tourism/When Netizens Asked Us to Make a Dragon Year Promo Video	C24	Shaoguan Culture & Tourism/Shaoguan, the Only City in China Named Shaoguan
C10	Shaoguan Culture & Tourism/Post-2000s Editor Still on Duty	C25	Haoran Lvi/I Found the Real “Dusty Inn” in Guangdong
C11	Coalball Hero/Shaoguan Is Such a Strange Yet Fun Place	C26	Mie A/Turns Out When Cantonese People Travel, They All Think About…
C12	Qiong Niangniang/When Will Guangdong’s Summer Ever End?	C27	Xiaohei Zhuming/Exploring Shaoguan Danxia Mountain by Land, Water, and Air
C13	Vincent Yusen/Life Is Meant to Be Enjoyed	C28	Planet No.42/Here’s How to Play When You Visit Danxia Mountain
C14	Mie A/So These Are the Traits of Guangdong Girls…	C29	Chen Cheng/You Must Visit Danxia Mountain at Least Once
C15	Mao Lin Coco/She Doesn’t Admit She’s from Shaoguan, and She Can’t Even Speak Hakka	C30	Tomas Rao Min/Today We Returned to the Long-Dreamed-of Hometown

### 3.4 Research conditions and outcome

This study employs the Heuristic–Systematic Model (HSM) to specify how multimodal cues in Douyin videos shape digital urban communication for Shaoguan, Guangdong. In HSM, heuristic processing relies on low-effort cues, whereas systematic processing engages high-effort evaluation of message content [18–19]. In China’s short-video ecology, we further treat platform-visible signals (e.g., hashtags, covers, creator badges, duration) as algorithmic heuristics and content-level elements (e.g., emotional resonance, narrative structure, camera language) as supports for systematic processing. This framing is consistent with research on Douyin/TikTok’s parallel platformization and Chinese short-video narratives [3,13]. We therefore model eight conditions: four heuristic (video duration, hashtags, cover visual design, creator attributes) and four systematic (content theme, emotional resonance, narrative structure, camera language).

Coding follows QCA best practices for necessity/sufficiency analysis in medium-N designs [24–25]. Two trained coders independently coded all cases; disagreements were resolved by discussion. By jointly operationalizing heuristic and systematic conditions, we tailor HSM to Chinese urban-communication contexts on Douyin rather than a generic “Asian” frame.

#### 3.4.1 Condition 1: Video duration.

Shorter clips reduce cognitive effort and favor heuristic uptake, whereas longer clips allow deeper meaning‑making—consistent with HSM’s motivation/ability account [18–19]. Empirically, duration is a salient content feature associated with engagement composition in short‑video feeds [26].

We code duration as a crisp set relative to the one‑minute norm of short‑form video: videos with runtime ≤ 60 seconds are coded 0 (Short Duration), and > 60 seconds coded 1 (Non‑Short Duration). This boundary aligns with common platform practices while leaving content‑level effects to be captured by other conditions.

#### 3.4.2 Condition 2: Hashtags.

Hashtags act as algorithmic and cultural signposts that index topics, guide recommendation, and provide quick semantic cues, thereby functioning as heuristics in Chinese short‑video contexts [3,13].

We record whether a video contains at least one city‑related or campaign/trending hashtag (e.g., #Shaoguan, #DanxiaMountain). Presence = 1; absence = 0. We also note tag specificity in Table 2 but retain a binary indicator for csQCA.

**Table 2:** Configuration of conditions and outcome.

Condition/Outcome	Category	Description	Share in sample	Assignment
**Heuristic** **Conditions**				
Video Duration	Short Duration	Videos ≤1 minute reduce cognitive load, aligning with fragmented browsing habits.	30.0%	0
	Non-Short Duration	Videos >1 minute require greater user attention.	70.0%	1
Hashtag	City-Related Hashtags	Videos with hashtags (e.g., #Shaoguan) enhance dissemination via algorithmic prioritization.	93.3%	1
	No City-Related Hashtags	Absence of relevant hashtags limits algorithmic reach.	6.7%	0
Cover Visual Design	Iconic Elements	Covers with iconic elements (e.g., Danxia Mountain) attract rapid user attention.	66.7%	1
	No Iconic Elements	Lack of distinctive visuals reduces initial engagement.	33.3%	0
Creator Profile	Local Creator	Local creators (e.g., Shaoguan residents) convey authentic city images.	23.3%	0
	Non-Local Creator	Non-local creators offer external perspectives and novelty cues, while they may have less embedded local knowledge.	76.7%	1
**Systematic** **Conditions**				
Content Theme	Single Theme	Focus on one theme (e.g., scenery, cuisine) creates clear narratives.	63.3%	1
	Composite Theme	Videos combine multiple themes (e.g., scenery + cuisine + history), broadening appeal while reducing single-theme focus.	36.7%	0
Emotional Resonance	Present	Videos evoking nostalgia or pride enhance city image identification.	86.7%	1
	Absent	Informational videos without emotional impact limit engagement.	13.3%	0
Narrative Structure	Coherent/Structured	Coherent narratives (e.g., storytelling) strengthen content integration.	40.0%	0
	Fragmented	Fragmented or loosely structured narratives align with mobile scanning but reduce narrative coherence.	60.0%	1
Camera Language	Distinctive	Professional techniques (e.g., aerial shots) enhance expressiveness.	26.7%	0
	Ordinary	Ordinary camera styles provide documentary immediacy but limited visual distinctiveness.	73.3%	1
**Digital Content Influence Outcome**	High Digital Content Influence	Cases above the median within the sampled high-interaction archive.	50.0%	1
	Low Digital Content Influence	Cases below the median within the sampled high-interaction archive.	50.0%	0

#### 3.4.3 Condition 3: Cover visual design.

Covers (thumbnails) are first‑impression cues. Salience, iconicity, and compositional clarity prime attention and expectations; these design factors shape downstream judgments and are well‑documented in visual/branding research [19,27].

We identify whether the cover features iconic/place‑specific elements (e.g., Danxia Mountain silhouette, landmark typography) with clear, high‑contrast composition. Presence of iconic/clear cover = 1; otherwise = 0. Examples and counter‑examples are shown in Table 2.

#### 3.4.4 Condition 4: Creator profile.

Creator profile conditions authenticity and novelty: local creators embed dialects and customs that foster identification, while non-local creators provide outsider perspectives that may generate short-term visibility--patterns observed in Douyin/TikTok scholarship and influencer video research [3,13,28].

We code creator profile using self‑descriptions and geotags: Local creator = 0; Non‑local creator = 1. Ambiguous cases (e.g., travel vloggers without declared origin) are adjudicated via profile/history and documented in Table 2.

#### 3.4.5 Condition 5: Content theme.

Themes structure cognitive processing: information‑rich or composite themes stimulate systematic processing; Chinese multimodal work shows city videos braid modernity and heritage to widen appeal [6,19].

We operationalize this condition by distinguishing single-focus themes from composite themes based on whether one domain dominates (e.g., only scenery) or multiple domains co‑occur (e.g., scenery + cuisine + history). Following the existing calibration used downstream, Single theme = 1; Composite theme = 0 (see Table 2).

#### 3.4.6 Condition 6: Emotional resonance.

Affective cues (awe, pride, nostalgia) increase sharing propensity and sustain identification; narrative‑emotion research further shows that emotional flow supports persuasive outcomes [14,29].

We code Emotion = 1 when videos contain explicit affective displays or cues (e.g., affect‑laden narration, expressive voice/music, on‑screen reactions) that foreground joy/pride/awe/nostalgia; otherwise 0. Coding anchors and examples are specified in Table 2.

#### 3.4.7 Condition 7: Narrative structure.

Fragmented vignettes align with mobile scanning (rapid, low‑effort decoding), whereas coherent storylines support identification and distinctiveness—consistent with narrative/encoding theory [16,30–33].

To preserve consistency with downstream calibration, we code Fragmented narrative = 1 (e.g., montages, slogan‑driven cuts without an integrating arc); Coherent/structured narrative = 0. Decision rules and examples are detailed in Table 2.

#### 3.4.8 Condition 8: Camera language.

Visual technique affects informational yield and engagement in city‑video contexts [7,34]. Distinctive techniques (e.g., drones, rapid edits) can signal production value; ordinary styles can index authenticity.

In line with the analysis that follows, we code Ordinary camera style = 1 (e.g., static/handheld with minimal effects) and Distinctive/professional techniques = 0 (e.g., aerials, hyperlapse). See Table 2 for illustrations.

#### 3.4.9 Outcome: Digital content influence.

Short‑video dissemination is tightly coupled with user engagement on the platform. A substantial body of work on Douyin/short‑video research treats likes, comments, saves (favorites), and shares as core, observable indicators of a video’s influence and audience participation [35–36].

In industry practice, Qingbo Intelligence’s Douyin leaderboard operationalizes communication performance with the Douyin Communication Influence Index (DCI, V1.0), defined as Publishing Index (10%) + Engagement Index (76%) + Reach Index (14%); within the Engagement Index, likes (17%)/ comments (37%)/ shares (46%) are further weighted, underscoring the primacy of engagement in dissemination assessment [37]. This emphasis aligns with platform‑side mechanisms: Douyin’s personalized recommendation system explicitly considers behavioral signals such as likes, comments, shares/forwards, favorites, and watch time to predict interest and amplify exposure [38]. Empirical studies also observe that early accumulation of likes, comments, saves, and shares increases the likelihood of subsequent recommendations and broader reach, producing viral‑like diffusion patterns [39].

To ensure replicability using publicly accessible Douyin video-level data, this study focuses on engagement and constructs an author-defined index, DCI*, as a tractable proxy for a single video’s dissemination influence. Because the archive consists of already visible videos, the low-outcome category refers to relatively lower dissemination performance within the sampled high-interaction archive, rather than to low visibility on Douyin as a whole.


$$\begin{array}{c} {\text{DCI}}^{*}\;=\text{0}.\text{40}\times \text{Likes}+\text{0}.\text{30}\times \text{Comments}+\text{0}.\text{20}\times \text{Saves }(\text{Favorites})+\text{0}.\text{10}\times \text{Shares} \end{array}$$

The weighting gives greater emphasis to immediately visible engagement signals, especially likes and comments, while retaining saves and shares as indicators of more deliberate engagement. This author-defined index is not intended to replicate Qingbo’s official DCI, but to provide a transparent and reproducible proxy based on publicly visible video-level data. Platform disclosures and algorithm-audit evidence jointly support the premise that engagement signals are central to recommendation-driven exposure [38–39].

### 3.5 Measurement and coding reliability

We binarized all conditions using a 0.50 membership threshold, consistent with set-theoretic logic and reporting conventions in csQCA [24–25]. Thresholds were anchored in the operational definitions reported in Table 2; cases at or above the stated boundary were coded 1 and the remaining cases were coded 0. Two trained coders independently coded the full sample using an HSM-informed codebook after pilot calibration. Intercoder reliability reached Cohen’s κ = 0.85, indicating substantial agreement [40]. Disagreements were resolved through adjudication and minor clarification of coding rules, and the reconciled dataset was used for downstream QCA.

### 3.6 Ethics statement

This study did not involve prospective recruitment, interventions, interviews, surveys, experiments, or access to private human-participant records. It analyzed publicly available Douyin short-video content, public account self-presentations/geotags used only to determine creator profile, hashtags, and publicly visible video-level engagement metrics. No private communications, restricted-access information, sensitive personal data, or non-public identifiers were collected. Informed consent was not obtained because the study did not recruit or interact with human participants, and no attempt was made to identify individuals beyond public account names or pseudonyms already displayed on the platform

## 4. QCA data analysis for small and medium-sized city image dissemination

### 4.1 Construction of the truth table

Following best practices for crisp-set Qualitative Comparative Analysis (csQCA), we calibrated eight conditions—video duration, hashtag use, cover visual design, creator profile, content theme, emotional resonance, narrative structure, and camera language—and one outcome, digital content influence, for 30 high-interaction Douyin videos about Shaoguan (May 2023–May 2025). Each case received binary membership (1 = presence, 0 = absence) according to the calibration rules in Table 2, and the resulting truth table (Table 3) maps condition configurations to the outcome, enabling the identification of causal pathways to effective city-image dissemination in Chinese small and medium-sized cities [24,25].

**Table 3:** Truth table.

Case	Duration	Hashtag	Visuals	Profile	Theme	Emotion	Narrative	Camera Language	Outcome
C1	0	1	0	1	1	1	1	0	1
C2	1	1	1	1	1	1	0	1	1
C3	1	1	0	0	1	0	0	1	1
C4	1	1	1	1	1	1	0	1	1
C5	1	1	1	1	1	1	0	0	1
C6	1	1	1	0	1	1	0	0	1
C7	1	1	1	1	0	1	1	1	1
C8	1	1	0	1	0	1	1	0	0
C9	0	1	1	0	1	0	1	1	1
C10	0	1	1	0	1	0	1	1	0
C11	1	1	1	1	0	1	0	1	0
C12	1	1	0	1	1	0	1	1	1
C13	1	1	1	1	1	1	1	0	1
C14	0	1	0	1	0	1	1	1	1
C15	1	1	1	1	0	1	1	1	1
C16	1	1	1	1	0	1	0	1	0
C17	0	1	1	0	1	1	1	0	0
C18	1	1	0	1	1	1	0	1	0
C19	1	1	1	1	1	1	1	1	0
C20	1	1	1	1	0	1	0	1	0
C21	1	0	0	1	0	1	1	1	0
C22	0	1	1	0	0	1	1	1	1
C23	1	1	1	1	0	1	1	1	1
C24	0	1	1	0	0	1	1	1	0
C25	0	1	0	1	1	1	1	1	0
C26	0	1	0	1	1	1	0	1	1
C27	1	1	1	1	1	1	1	0	0
C28	1	1	1	1	1	1	0	1	0
C29	1	1	1	1	1	1	0	0	0
C30	1	0	0	1	1	1	1	1	0

### 4.2 Necessity analysis of single conditions

The necessity analysis indicates that Hashtag is the only condition exceeding the conventional 0.90 consistency threshold. In the sampled archive, all high-dissemination cases contain city-related hashtags, whereas no high-dissemination case occurs without them. Given the high prevalence of hashtags in the sample, this finding should be interpreted as a necessary platform-indexing condition rather than as a standalone driver of dissemination performance. Full necessity results are reported in Table 4.

**Table 4:** Necessity analysis of single conditions.

Condition	Consistency	Coverage	RoN
Duration	0.666667	0.476190	0.45
~Duration	0.333333	0.555556	0.31
Hashtag	1.000000	0.535714	**0.72**
~Hashtag	0.000000	0.000000	0.00
Visuals	0.666667	0.500000	0.38
~Visuals	0.333333	0.500000	0.29
Profile	0.733333	0.478261	0.40
~Profile	0.266667	0.571429	0.33
Theme	0.666667	0.526316	0.41
~Theme	0.333333	0.454545	0.27
Emotion	0.800000	0.461538	0.55
~Emotion	0.200000	0.750000	0.28
Narrative	0.600000	0.500000	0.36
~Narrative	0.400000	0.500000	0.32
Camera Language	0.733333	0.500000	0.43
~Camera Language	0.266667	0.500000	0.30

**Fig 1:**
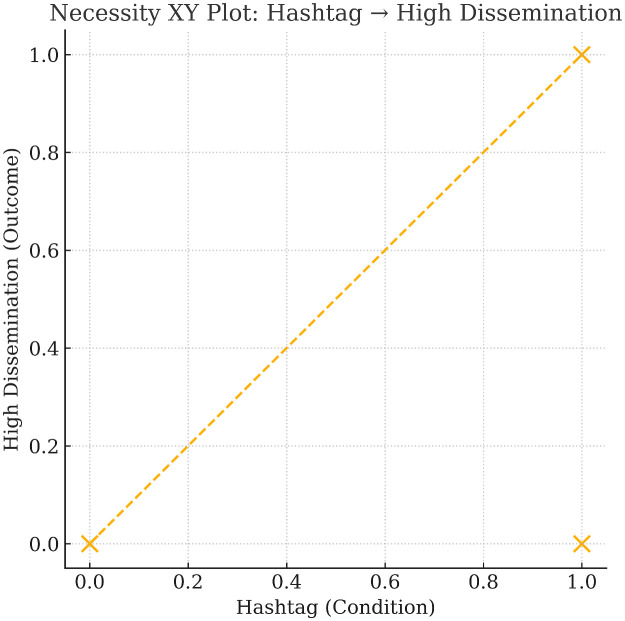
XY plot of hashtag as a necessary condition.

Table 5 and Fig 1 further confirm that no high-dissemination case lacks city-related hashtags, supporting the interpretation of hashtag use as a necessary platform-indexing condition in this sample.

**Table 5:** Necessity analysis of hashtag condition.

Condition	Consistency	Coverage	RoN	Deviant Cases for Necessity
Hashtag	1.000	0.536	0.72	None
~Hashtag	0.000	0.000	0.00	--

### 4.3 Sufficiency analysis of condition configurations

To uncover configurational pathways leading to high dissemination performance of Shaoguan’s city image on Douyin, we analyzed the calibrated dataset with fsQCA 4.1 and constructed a truth table for cross-case comparison. Following best practice for small-N designs, we set frequency cutoff = 1 to retain rare but potentially informative combinations, and consistency cutoff = .80, a widely used benchmark in QCA applications [25,41]. Because Hashtag was established as necessary in Section 4.2, we fixed Hashtag = 1 during minimization; the remaining seven conditions (duration, cover, creator profile, theme, emotion, narrative, camera) were treated as dichotomous.

We estimated the complex, parsimonious, and intermediate solutions. The complex solution preserves all observed configurations without minimization, reflecting the multidimensionality of short-video communication. The parsimonious solution yields highly simplified expressions but risks overlooking context. We adopt the intermediate solution—which incorporates theory-informed directional expectations while excluding implausible counterfactuals—as it best balances empirical fit and theoretical plausibility [24,42,43]. The intermediate solution returns seven sufficient pathways (see Table 6), with solution coverage = 0.60 and solution consistency = 1.00.

**Table 6:** Qualitative comparative analysis results.

Condition Configuration	Raw Coverage	Unique Coverage	Consistency	PRI Consistency
DUR * HAS * ~ VIS * ~ PRO * THE * ~ EMO * ~ NAR * CAM	0.067	0.067	1.00	0.85
DUR * HAS * VIS * ~ PRO * THE * EMO * ~ NAR * ~ CAM	0.067	0.067	1.00	0.86
~DUR * HAS * ~ VIS * PRO * THE * EMO * NAR * ~ CAM	0.067	0.067	1.00	0.83
~DUR * HAS * ~ VIS * PRO * THE * EMO * ~ NAR * CAM	0.067	0.067	1.00	0.84
~DUR * HAS * ~ VIS * PRO * ~ THE * EMO * NAR * CAM	0.067	0.067	1.00	0.82
DUR * HAS * ~ VIS * PRO * THE * ~ EMO * NAR * CAM	0.067	0.067	1.00	0.81
DUR * HAS * VIS * PRO * ~ THE * EMO * NAR * CAM	0.200	0.200	1.00	0.88
**Solution Coverage**	**0.60**		**1.00**	

To guard against spurious findings, we report PRI-consistency (Proportional Reduction in Inconsistency), which gauges the extent to which each configuration avoids simultaneously covering negative cases [25]. All retained configurations achieve PRI ≥ 0.80, comfortably above the conventional 0.65 threshold for sufficiency claims in configurational research [44].

Substantive interpretation. Two observations stand out.

First, the pathway with the largest unique coverage (Duration * Hashtag * Visuals * Profile * ~ Theme * Emotion * Narrative * Camera Language) indicates an immersive diffusion pattern. In this configuration, longer duration, iconic covers, non-local creators, composite themes, affective resonance, and fragmented storytelling combine to make Shaoguan legible and engaging within mobile feeds. This result supports the view that platform visibility is produced by bundles of cues rather than by any single content feature [3,7,17].

Second, smaller-coverage pathways show equifinality. Coherent narration can substitute for distinctive cinematography in some single-theme videos, while affective and visual cues can compensate for weaker narrative integration in others. The findings therefore point to multiple viable cue-narrative combinations for SMC city-image dissemination.

From an HSM perspective, fixing Hashtag = 1 in all sufficient paths confirms the heuristic role of platform indexing, while emotion and narrative structure explain how meaning and identification are sustained. The results thus connect algorithmic visibility with cultural interpretation rather than treating dissemination as a purely technical outcome.

### 4.4 Robustness inspection

To assess the reliability of the intermediate solution (solution coverage = 0.60, solution consistency = 1.00), we implemented multiple robustness checks recommended for small-N csQCA. First, we raised the consistency cutoff from 0.80 to 0.90 [25]. The re-estimated truth table preserved the core pathways, indicating that our findings are not sensitive to moderate calibration tightening. Second, we conducted a random case-deletion test by re-running the model on a 25-case subsample; the pathway with the largest unique coverage (Path 7) remained, while several very low-coverage paths disappeared as expected under reduced diversity. Third, we excluded the four cases with Emotion = 0 (13.3% of N = 30); Path 7 again persisted, underscoring emotional resonance as a stable contributor to high dissemination performance.

Consistent with best practice for datasets of this size, we set frequency cutoff = 1 to retain rare but potentially informative configurations; raising it to 2 would unduly prune empirically meaningful combinations in a 30-case design [24,25,41]. To avoid spurious sufficiency claims driven by simultaneous subset relations, we report PRI-consistency in addition to raw consistency [25]. As shown in Table 6, all retained configurations achieve PRI ≥ 0.80, comfortably above the commonly used 0.65 benchmark in configurational research [44].

Given eight conditions and 30 cases, limited diversity is expected—many logically possible configurations remain unobserved [24,25]. In the intermediate solution, we therefore borrowed a subset of logical remainders under theory-guided directional expectations (DEs) [42,43]: configurations with HASHTAG = 1 and EMOTION = 1 were assigned positive directional expectations, consistent with the HSM view of heuristic (hashtags) and systematic (emotion) synergy. Sensitivity tests that relaxed DEs for Narrative or Camera left the core pathway intact, indicating that our conclusions are not an artifact of a particular remainder-borrowing scheme. (See Table 7.)

**Table 7:** Borrowed logical remainders and theory‑guided directional expectations (DEs).

Configuration (Simplified)	Borrowed?	Directional Expectation (DE)	Theoretical Justification
HAS * EMO	Yes	Positive → High Dissemination Performance	Consistent with HSM: synergy of heuristic (hashtags) and systematic (emotional) cues should increase impact.
HAS * ~ EMO	No	—	For unobserved remainders, hashtag use without emotional resonance was not assigned a positive directional expectation, because heuristic indexing alone does not theoretically guarantee deeper engagement.
~HAS * EMO	No	—	Emotional resonance without topical indexing is less likely to achieve wide dissemination performance.
HAS * ~ NAR * ~ CAM	Yes	Positive → High Dissemination Performance	Coherent storytelling with distinctive camera techniques plus accurate hashtags is expected to enhance systematic processing.
~HAS	No	—	Inconsistent with the necessity test: hashtag absence cannot be assumed to produce high dissemination performance.

This table lists unobserved configurations evaluated at the intermediate QCA stage and indicates whether a remainder was borrowed, the assigned directional expectation toward the outcome (→ High Dissemination Performance), and the theoretical rationale. Guided by the Heuristic-Systematic Model (HSM), remainders combining hashtag use with emotional content (HAS * EMO) were assigned a positive directional expectation, reflecting the joint effect of heuristic and systematic cues. Configurations combining hashtag use with coherent storytelling and distinctive camera techniques were also assigned a positive directional expectation. Given the calibration used in this study, this latter configuration is expressed as HAS * ~ NAR * ~ CAM, where ~NAR denotes coherent/structured narrative and ~CAM denotes distinctive/professional camera language. In contrast, remainders without hashtags (~HAS) or without emotional resonance (~EMO) were not borrowed, as doing so would contradict the necessity analysis and the underlying theory. Sensitivity checks with alternative directional expectations did not alter the core solution, indicating that results are not dependent on a particular remainder specification.

Notes: “*” denotes logical AND; “~” denotes absence (0); “other conditions varied” indicates that remaining conditions may take any value. “Borrowed” marks logical remainders incorporated into the intermediate solution under the stated DE. In this study, NAR = 1 denotes fragmented narrative and CAM = 1 denotes ordinary camera language; therefore, ~ NAR indicates coherent/structured narrative and ~CAM indicates distinctive/professional camera techniques. Abbreviations: DE = Directional Expectation; HSM = Heuristic-Systematic Model.

Taken together--threshold elevation, random resampling, targeted case exclusion, PRI reporting, and transparent treatment of logical remainders--these checks support the relative stability of the identified pathways across calibration changes, sample variation, and alternative counterfactual assumptions.

## 5. The salient high-impact pathway: Immersive diffusion

**Fig 2:**
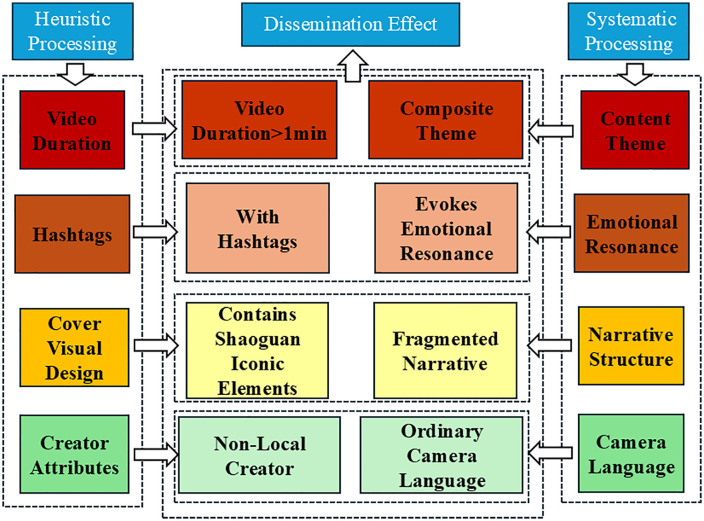
Immersive diffusion model.

Path 7 (Duration * Hashtag * Visuals * Profile * ~ Theme * Emotion * Narrative * Camera Language)—the pathway with the largest unique coverage—features longer videos (>60s), Shaoguan-related hashtags, iconic covers, non-local creators, composite themes, affective resonance, fragmented storytelling, and ordinary camera language. We interpret this as an “immersive diffusion” pattern (see Fig 2): longer duration enables multi-scene montage that compensates for non-distinctive cinematography; composite themes raise cognitive variety yet are held together by emotion and bite-sized narrative beats. This is consistent with dual-process accounts in which heuristic cues (e.g., tags, thumbnails) capture attention, and systematic elements (emotion, narrative structure) sustain meaning [18,19].

Two illustrative Douyin videos embody this immersive diffusion. One by user Los Angeles Yingzheng showcases Danxia Mountain and local cuisine through fragmented yet humor-laden sequences, conveying Shaoguan’s natural and cultural appeal. Another by Xu Ge Yuan interweaves scenes of regional culinary heritage, Danxia’s landscapes, and Shaoguan’s historic East Street into a sensory collage that stimulates sensory-rich viewer engagement. These narratives, aided by emotional resonance, leverage algorithmic visibility to enhance dissemination potential. In both, hashtags foreground topical indexing while emotion and fragmentation sustain scrolling-context engagement--an arrangement aligned with recent work on content logics in Douyin/TikTok’s parallel platformization [3].

### 5.1 The “Traffic Attraction–Cultural Resilience” dual-helix model

**Fig 3:**
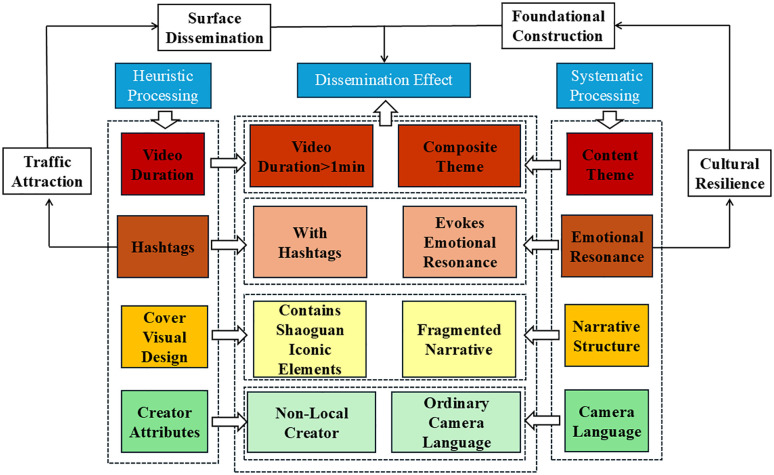
Dual-helix structure of dissemination.

Within China’s platformized short-video ecology, city-image communication hinges on a dual logic: short-term attention capture (algorithmic exposure via heuristic cues) and long-term cultural resilience (identity-rooted meaning via systematic processing). Applying csQCA to Shaoguan shows how small and medium-sized cities can balance fleeting visibility with enduring identity. This dual-helix model, illustrated in Fig 3, reconciles creator-driven ephemeral attention with deeper narrative rootedness. It offers a potentially transferable framework for comparable small and medium-sized cities in platformized short-video environments.

### 5.2 Traffic attraction: Algorithm-driven dissemination and emotional engagement (see Fig 4)

Content embedded with event/locale-specific hashtags plus affective cues aligns with Douyin’s recommendation logic. Tags (e.g., #ShaoguanTravelGuide) act as algorithmic heuristics that index topics and audiences, while affective cues sustain viewer engagement and facilitate place-based identification. Empirical research documents tags’ privileged role within Douyin/TikTok’s platformization, and feature-level analyses of city-image videos show how content attributes map onto engagement bundles [3,7,13]. These dynamics turn ephemeral attention into durable city-image communication through word-of-mouth and networked sharing.

**Fig 4:**
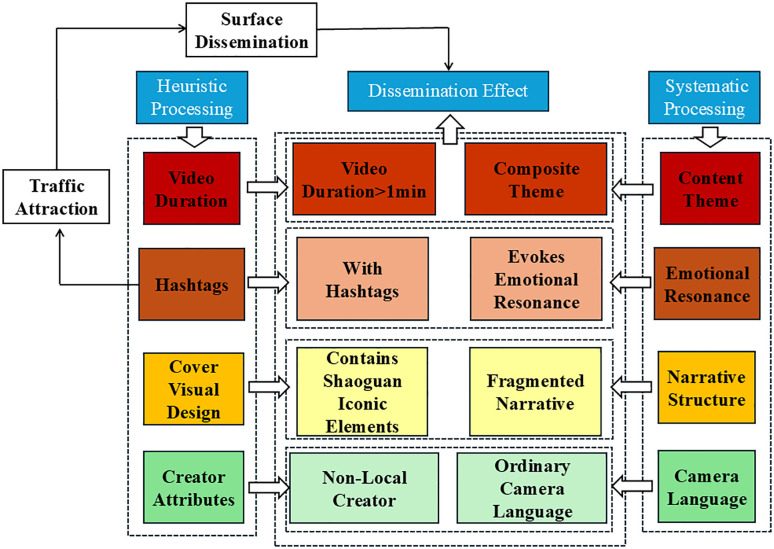
Traffic attraction – surface dissemination.

Case evidence suggests that decomposing a city image into recognizable cognitive units—iconic landmarks and everyday practices—accelerates reach. Yet surface imagery alone risks low retention; layering emotion and micro-narratives mitigates this risk and strengthens identification.

### 5.3 Cultural resilience: Deep narratives and cognitive engagement (See Fig 5)

Beyond traffic, cultural resilience depends on layered storytelling and meaning reconstruction. Non-local creators often act as “cultural decoders,” reframing urban narratives through outsider lenses; local creators anchor embedded knowledge and coherent narratives that reinforce place identity. Multimodal work on Chinese promotional videos similarly shows that deep narrative structures, sometimes with professional camera language, sustain long-tail engagement and place-based identification [6,7]. For SMCs, resilience is not only a creative task but also institutional curation: e.g., building a “narrative resource bank” to systematize local knowledge into reusable modules for short-video storytelling.

**Fig 5:**
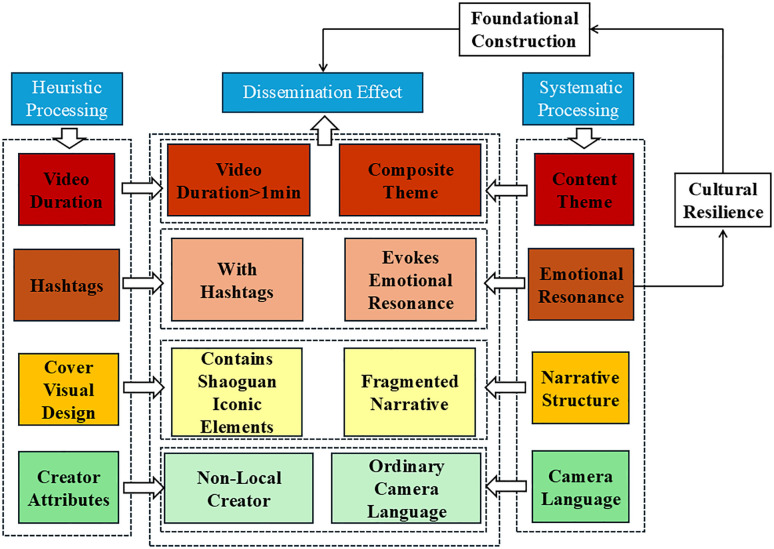
Cultural resilience – foundational construction.

## 6. Discussion

The findings provide empirical grounding for the three theoretical propositions developed in this study, while also refining them in configurational terms. Shaoguan’s high-dissemination Douyin videos are not driven by a single universal formula. Instead, visibility emerges from configurations that combine algorithmic heuristics with affective, narrative, and positional resources. Proposition 1 is refined by the finding that hashtag presence operates as a necessary platform-indexing condition in the sampled archive, although its moderate coverage suggests that it should be interpreted as enabling rather than sufficient on its own. Visual packaging works less as an isolated driver than as a platform-addressability cue that becomes effective within broader content configurations. Proposition 2 receives clearer configurational support: high dissemination performance is more likely when rapid recognizability is coupled with emotional resonance and culturally meaningful narrative structures. Proposition 3 is also specified: non-local creators can generate novelty, contrast, and external attention, whereas local creators can strengthen embedded knowledge, cultural continuity, and place-based interpretation. Taken together, these findings show that short-video visibility for small and medium-sized cities depends not merely on attention capture, but on the coupling of traffic-oriented cues with culturally grounded meaning-making.

This also explains why Shaoguan’s visibility on Douyin should not be understood simply as the result of algorithmic amplification. Hashtags and visual cues help local symbols enter searchable and recommendable attention flows, but they do not automatically produce sustained communication effects. Dissemination becomes stronger when platform cues are paired with emotional resonance, creator positioning, and narrative structures that help viewers recognize Shaoguan as both a destination and a culturally meaningful place. In this sense, platform visibility is not only a technical outcome of indexing and recommendation, but also a cultural process through which place meanings are selected, packaged, circulated, and reinterpreted.

### 6.1 Theoretical implications

The study contributes to theory in three ways. First, it extends the heuristic-systematic model from a general model of persuasive processing to a platform-specific account of city-image communication. In short-video ecologies, heuristic cues such as hashtags, covers, visual symbols, and recognizable scenes help content gain initial attention, while systematic resources such as affective resonance, narrative organization, and cultural explanation sustain deeper place recognition. This extension shows that HSM can be used not only to explain individual information processing, but also to conceptualize how platform-native visibility is organized through the interaction between quick cues and culturally meaningful content.

Second, the study connects digital place branding with platformization research by demonstrating that city brands are not simply authored by municipal institutions. Rather, they are co-produced through creator labor, platform indexing, user engagement, and algorithmic visibility [2,4,5]. For small and medium-sized cities, this is especially important because their visibility often depends less on large-scale official campaigns than on dispersed creator practices and platform-mediated circulation. The case of Shaoguan shows that city image is increasingly shaped through a hybrid process in which official cultural resources, local memories, outsider curiosity, and platform logics are intertwined.

Third, the configurational findings move beyond linear explanations by showing that different cue-narrative-position bundles can produce similar dissemination outcomes. This is especially important for resource-constrained small and medium-sized cities because it suggests that city-image visibility does not depend on one optimal communication formula. Instead, different combinations of platform heuristics, creator positionality, emotional resonance, and narrative organization can generate comparable levels of digital content influence. The broader theoretical implication is that short-video city communication should be understood through a configurational logic: what matters is not whether a single factor is present, but how multiple cues and cultural resources are assembled into a recognizable and circulable form.

These implications are captured by the Traffic Attraction-Cultural Resilience dual-helix model proposed in this study. The traffic-attraction dimension refers to the platform-facing work of making the city searchable, recognizable, and recommendable. The cultural-resilience dimension refers to the place-facing work of preserving, renewing, and narratively organizing local cultural meaning. The two dimensions are not mutually exclusive. Effective city communication on short-video platforms requires their interaction: cities must enter attention flows through platform-native cues, but they must also sustain place identity through affective and narrative depth.

### 6.2 Practical implications

The findings offer practical implications for municipal communicators, cultural institutions, and local creators in China.

(1) Standardize a hashtag taxonomy that combines official cultural identifiers (e.g., #ShaoguanHakka) with topical/trending descriptors to improve algorithmic addressability [3,13].
(2) Co-produce with creator diversity: pair non-local creators (novelty/contrast) with local creators (embedded knowledge) to avoid echo chambers and widen reach [3].
(3) Modularize narrative resources: curate a narrative resource bank (heritage episodes, symbolic landmarks, everyday practices) and couple emotion + micro-story beats to convert exposure into recognition [6–7].
(4) Align design with attention ecology: use iconic covers and clear visual cues to capture attention during scrolling, then sustain attention with coherent story arcs consistent with the dual-helix logic.

For small and medium-sized cities, the practical lesson is therefore not simply to imitate the traffic strategies of major cities or popular tourist destinations. Instead, they need to build a communication system that links platform readability with local cultural depth. A successful short-video strategy should make local culture easy to find, easy to recognize, and easy to circulate, while also ensuring that the city is not reduced to a set of superficial visual symbols.

### 6.3 Limitations and future research

Several limitations should be acknowledged. First, DCI* is an author-defined composite indicator based on publicly visible engagement metrics. Although it is grounded in platform-disclosure logic and short-video research, alternative weighting schemes may produce different sensitivity patterns. Future research could compare different outcome functions and weighting strategies to test the stability of the results.

Second, the study focuses on one city and one platform. Shaoguan provides a useful case for examining the platform visibility of a non-metropolitan Chinese city, but the findings cannot be automatically generalized to all small and medium-sized cities. Future studies could replicate the model across multiple Chinese cities, different regional cultures, and different types of urban identity.

Third, the archive captures videos that had already achieved relatively high interaction. Therefore, the analysis explains configured visibility within an existing attention circuit rather than predicting virality for all city-related videos. Future research could include lower-engagement cases or longitudinal data to examine how videos move from initial exposure to wider dissemination.

Fourth, this study uses crisp-set QCA to identify interpretable configurations. This approach is appropriate for clarifying the presence or absence of key conditions, but it may compress fine-grained differences among cases. Future research could compare crisp-set results with fuzzy-set calibrations, alternative thresholds, or mixed-method designs to examine the sensitivity of the Traffic Attraction-Cultural Resilience dual-helix model.

Future research can extend the analysis in four directions: replicating the model across multiple Chinese small and medium-sized cities and subcultures; comparing Douyin with platforms such as Bilibili, Xiaohongshu, or WeChat Channels; connecting online engagement with offline indicators such as tourist footfall, bookings, or cultural-event participation; and testing alternative calibration thresholds and outcome functions to examine the sensitivity of the dual-helix model.

### 6.4 Conclusion

This article examined how Chinese small and medium-sized cities can achieve city-image visibility on Douyin. By integrating the heuristic-systematic model with crisp-set QCA, implemented with fsQCA 4.1 for Windows, it identified hashtag presence as a necessary platform-indexing condition and uncovered seven sufficient pathways to high digital content influence. Returning to the three theoretical propositions, the findings show that platform heuristics are enabling rather than sufficient conditions: hashtags and visual packaging help local cultural symbols enter algorithmic attention flows, but high dissemination performance depends on how these cues are configured with emotional resonance, narrative organization, and creator positionality.

The most salient pathway, immersive diffusion, combines algorithmic readability with affective and fragmented storytelling, suggesting that platform visibility is produced through both immediate recognizability and culturally meaningful engagement. The study’s broader contribution is the Traffic Attraction-Cultural Resilience dual-helix model, which explains how short-video city communication can balance immediate platform traffic with longer-term cultural meaning. For small and medium-sized cities, the central challenge is not simply to be seen, but to be represented in ways that preserve and renew place identity beyond momentary exposure.

### Code availability

No custom programming scripts, macros, or author-generated software were used. The fsQCA analyses were performed using fsQCA 4.1 for Windows. The author-defined DCI* outcome score was calculated using transparent Excel formulas embedded in the deposited dataset: DCI* = 0.40 × Likes + 0.30 × Comments + 0.20 × Saves (Favorites) + 0.10 × Shares.
